# The year 2020, a milestone in breaking the vicious cycle of poverty and illness in China

**DOI:** 10.1186/s40249-020-0626-5

**Published:** 2020-01-30

**Authors:** Yun-Ping Wang, Xiao-Nong Zhou

**Affiliations:** 1 China National Health Development Research Centre, National Health Commission of China; WHO Collaborating Centre for Health Systems Strengthening, Beijing, 100044 China; 2 National Institute of Parasitic Diseases at Chinese Center for Diseases Control and Prevention; Chinese Center for Tropical Diseases Research, Shanghai, 200025 China; 3 WHO Collaborating Centre for Tropical Diseases; National Center for International Research on Tropical Diseases, Ministry of Science and Technology; Key Laboratory of Parasite and Vector Biology, Ministry of Health, Shanghai, 200025 China; 4 School of Global Health, Chinese Center for Tropical Diseases Research, Jiatong University School of Medicine, Shanghai, 200025 China

**Keywords:** Poverty alleviation, Infectious diseases, Poor population, Health insurance, Endemic diseases, China, Healthy China

## Abstract

Marking the end of the five-year programme initiated by the Chinese Government to lift more than 70 million people out of poverty, the year 2020 is a milestone. Poverty alleviation has moved strongly forward in China and the major health indicators are now better than the average of all middle- and high-income countries. However, the dual burden of infectious and chronic diseases remains a challenge with respect to achieving the health target in the United Nations 2030 Agenda for sustainable development goals (SDGs). In 2015, about 44% of the poor population in China were impoverished by illness but already in 2018, multi-sectoral actions delivered by the Health-related Poverty Alleviation programme had reduced the number almost by half. In the past three years 15 million poor people (98% of the poor population) with infectious and chronic diseases had been treated and taken care of thanks to financial support through multiple health insurance schemes and other governmental subsidies. This article discusses the lessons learnt with regard to health-related poverty alleviation in China with special reference to those still remaining impoverished by illness. Consolidation of the achievements reached and provision of basic needs to those still disadvantaged and in poor health will require a major improvement of accessibility to, and affordability of, health services. The next step towards enhanced productivity and better living conditions will involve upgrading of the capacity of health professionals in the poor regions, promotion of coherent efforts in health-related poverty alleviation and rural revitalization measures. As an additional measure, data monitoring and research on health poverty alleviation should be strengthened as they are essential to generate the evidence and knowledge needed to support the move in the direction envisioned by the SDGs, and the new Healthy China 2030 programme.

## Background

Ending poverty in all its forms everywhere is the first goal of the 17 Sustainable Development Goals (SDGs) of the United Nations 2030 Agenda [1]. This goal, promising to leave no one behind [2], recognizes that ending poverty is the greatest of the global challenges ahead [3], since 10% of the world’s population still live at or below USD 1.90 a day (the internationally agreed poverty line). Although this figure is down from 36% in 1990 to 16% in 2010 in China [4–6], the goal is still far away. Efforts to reduce poverty in association with poor health due to major communicable afflictions such as HIV/AIDS, tuberculosis, malaria, hepatitis and neglected tropical diseases (NTDs), as well as maternal mortality, preventable deaths of newborns and children less than 5 years old [2], were already part of the Millennium Development Goals (MDGs), which have now been supplanted by the SDGs. Indeed, poverty is the greatest adversary in relation to health in the developing countries and current evidence illustrates that good health is not only an outcome, but an essential component of poverty reduction [7].

Not that long ago, China had the world’s biggest poor population, but has made spectacular progress in reducing health-related poverty [8] and became already the first country to meet the MDG targets for reduction of the number of poor people by 2015 [9]. Over 700 million people, amounting to 70% of the world’s total poor, have been lifted above China’s national poverty line since 1978, the time when the country opened up and started economic reform [10]. As of 2014, China still had 70.2 million people in the rural areas living below the poverty line [8]. This population was identified and selected as target for a programme referred to as the *“**Decision of the Central Committee of Communist Party of China and the State Council on Winning the Tough Battle against Poverty**”* [11]. In 2015, the President of China Xi Jinping pronounced that “a well-off society is for all people in China, no one should be left behind - over next five years, China will lift all its current 70 million living beneath the poverty line to safety, which is an important step in implementing development agenda after 2015 achieving China’s two centenary goals for development” [12]. With this he set in motion the fight against health-related poverty, one of the top priorities of the programme mentioned above. However, it is now clear that the two centenary goals cannot be reached in entirety without successful efforts to alleviate and eventually eradicate extreme poverty [13].

**Fig. 1:**
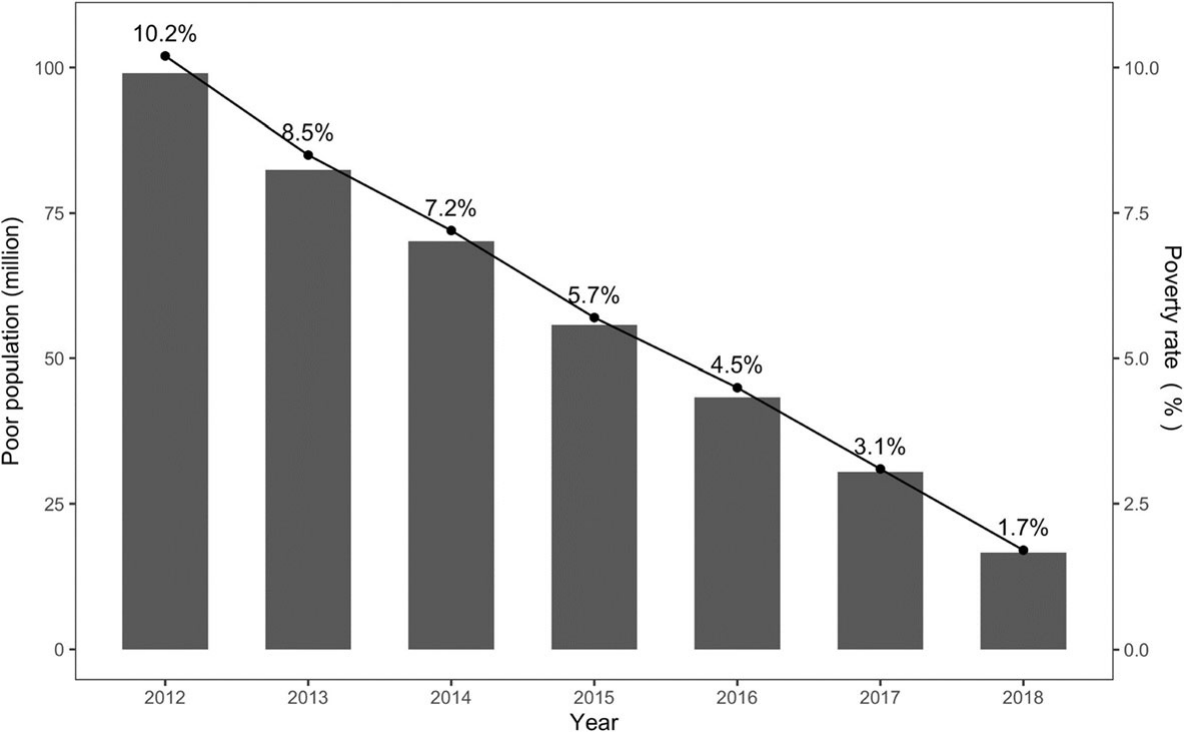
The progress in China’s poverty alleviation programme from 2012 to 2018. (Date source: National Bureau of Statistics, Statistical Bulletin of the National Economic and Social Development, 2012–2018)

According to the latest official data published by the Chinese Government, the rural poor population has been reduced by 80 million in total, which corresponds to an annual average decline of around 12 million people. Meanwhile, the poverty incidence rate declined at an annual rate of 13.8% (Fig. 1) receding from 10.2% in 2012 to 1.7% in 2018. By the end of 2019, an estimated 95% of the poor population around 11.09 million had been lifted out of poverty [12, 14]. Furthermore, the overall health status of the Chinese people has now become better than the average of the middle- and high-income countries in the past 5 years. For instance, from 2015 to 2018, the average life expectancy increased from 76.34 to 77 years, the infant mortality declined from 8.1‰ to 6.1‰, the under-5 mortality rate dropped from 10.7‰ to 8.4‰ and the maternal mortality rate fell from 20.1 to 18.3 per 100 000 [15].

Three basic questions are up-front when looking at available evidence-based data from China:

1. What is the connection between poverty and illness?
2. How can the vicious cycle of poverty and illness be defeated?
3. Which are the major lessons to be learnt from the health-related poverty alleviation programme?

## Poverty leads to illness

Health affects national economic growth because people with disease or disability are likely to be less productive at work, lose their jobs, retire or die prematurely. Naturally, all of these outcomes decrease household earnings and increase the risk of poverty. Hence, the foregone reduction of national income due to illness is considerable in countries where poor health is common. For example, the projected cumulative global loss of economic output due to non-communicable diseases (NCDs) from 2011 to 2030 is estimated at USD 47 trillion, with the joint share of the low and middle income countries (LMICs) amounting to around USD 21.3 trillion (46%) [16]. In addition, the cost for treatment of major infectious diseases, such as those mentioned above and/or increased microbial resistance to therapeutic drugs incur a financial shortfall for affected individuals and households. Out-of-pocket payments trap poor and near-poor households in a vicious cycle due to large personal expenditures leading to impoverishment and worse health, particularly in the LMICs where social health insurance schemes are generally lacking. Besides the NCDs and major infectious diseases, the NTDs constitute a group of diseases in tropical environments closely associated with poverty proliferation [9, 17]. Although many countries have made progress in the elimination of NTDs, an estimated 1.5 billion people in 2016 still require treatment and care due to affliction with one or more NTDs, among them about 400 million (27%) in low-income countries [4], emphasizing the worldwide presence of poverty and inequality. Every year, a reportedly 100 million people globally are pushed into poverty, often due to illness and pre-existing sickness aggravated by lack of essential health services [18].

As a large proportion of illnesses in the developing countries are entirely avoidable or treatable with existing medicines or interventions, disease burdens in these countries are often due to consequences of poverty, including poor nutrition, indoor air pollution and lack of access to proper sanitation and health education.

Historically, China has spent far too little on health, partly because health has not been seen as a “productive” part of the economy and also because of a previously low national income [19]. One of the lessons learnt was the disastrous collapse of Rural Cooperative Medical Scheme (RCMS), a community-based voluntary medical assistance scheme in the 1980s, which led to more than 800 million rural Chinese losing health care coverage in the following two decades [20, 21]. Some of the rural households are impoverished and discontent due to increasing medical expenses and long-term health care costs that could not be afforded.

**Fig. 2:**
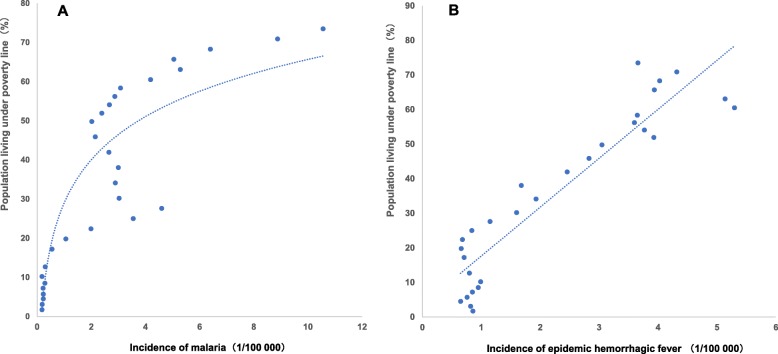
Poverty incidence and incidence of malaria (**a**) and epidemic hemorrhagic fever (**b**) in China (1990–2018) [15]. (**a** Spearman’s rank correlation coefficient of poverty incidence and malaria incidence is 0.88 (*P* < 0.01). **b** Spearman’s rank correlation coefficient of poverty incidence and epidemic hemorrhagic fever incidence is 0.89 (*P* < 0.01). Data source: Poverty incidence data is from World Bank database; and the incidence data of malaria & epidemic hemorrhagic fever is from China Health Statistics Yearbook 2019)

Alerted by several public focus events related to unaffordability of health expenditure, and the outbreak of the severe acute respiratory syndrome (SARS) in 2003, the Chinese Government launched the rural New Cooperative Medical Scheme (NCMS) for all the rural residents and rural Medical Financial Assistance Scheme (MFAS) for the rural poor to cover their health expenditure that year, and implemented a new health care reform in 2009, promising RMB 850 billion (USD 123 billion) over 3 years to provide universal health coverage, strengthened health services delivery and drug supply for its population then amounting to 1.3 billion [21, 22]. Currently, more than 95% of the Chinese population is covered by social health insurance schemes [23], and the percentage of people who needed, but did not receive, hospital-based treatment due to financial hardship decreased from 29.6% in 2003 to 17.1% in 2013 [24]. This ignores those who seek treatment but fall into poverty as a result of expenditure. However, this kind of poverty-related lack of proper care is still a challenge in China and to eliminate infectious diseases among the poor and improve their accessibility and affordability with respect to disease prevention, treatment and rehabilitation services will take more time [25]. For example, the results from three rounds of a national survey of important parasitic diseases showed that with the economic development, the average prevalence rate of soil-transmitted helminthiasis dropped from 59.8% in 1991 to 19.6% in 2003, and continued down to 4.8% in 2013 [9]. Another example is malaria, a disease with high mortality that traps households in poverty in many countries with a high burden of this infection [26–28], where Chinese evidence shows a significant Spearman’s rank correlation coefficient correlations between poverty and incidence of malaria (0.88, *P* < 0.01), as well as between poverty and epidemic hemorrhagic fever (0.89, *P* < 0.01) for the years 1990–2018 [15, 29–31] (Fig. 2). Among the poor in China, more than 44% are still impoverished by illness, such as cancer, childhood leukaemia, congenital heart disease, end-stage renal disease and infectious diseases, tuberculosis and parasitic diseases in particular [33].

## Defeating the vicious cycle

For the national poverty alleviation policy announced by the Chinese Government in 2015, the key measures include: (i) establishing a long-term mechanism for poverty relief and wealth acquisition; (ii) strengthening of infrastructure and basic public services in poor regions; and (iii) support for development of local industries and economy. The programme, identified as a priority in the overall framework to roll back poverty, has a five-area focus: (i) improving access to essential health services covered by health insurance and financial assistance schemes; (ii) strengthening health infrastructure and service delivery capacities in poor and rural regions; (iii) providing educational and training opportunities including attractive recruitment and retaining policies for the health workforce; (iv) promoting infectious and endemic disease elimination; and (v) supporting maternal and child health and nutrition in poor regions. These activities proved effective in responding to health-related poverty and 6.7 million households have been lifted out of the trap of impoverishment due to illness [23].

Success in fighting health-related poverty can be attributed to a two-pronged approach: i) strong political commitment and substantial investment from the government at all levels; and ii) appropriate technical strategies for improving health care and public health for the poor [8]. At the political level, the central government convened a national health conference in Beijing in 2016 to promote “Healthy China 2030”, a new domestic, cross-sectoral, long-term strategy to support global health and health-related SDGs with the slogan “Healthy lives and well-being for all” that was also used to continue the efforts combating emergence or re-emergence of infectious diseases. Given the universal and multi-sectoral nature of health, there is an urgent need to elevate work towards health to a higher level of priority and importance in many national contexts. The “Shanghai Declaration on Promotion of Health in the 2030 Agenda” reinforces good governance at all levels and is crucial for improving health-related matters [33, 34], which require investment and action at the local, national and also global levels. Thus, health is perceived as a crucial entry-point to achieving the SDGs because of its ability to lift people out of poverty making it central for individual, household and national socioeconomic development. Health is also is a critical component of human capital contributing to employability of people and general economic productivity.

**Fig. 3:**
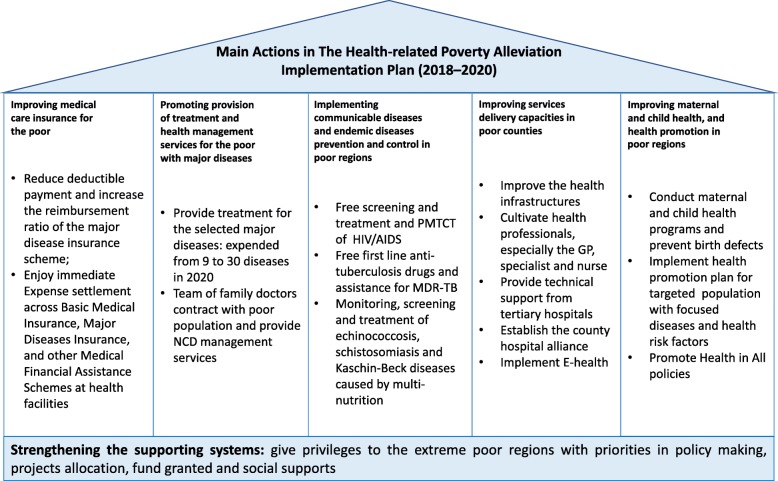
The diagram of the Main Actions of Health-related Poverty Alleviation Programme in China. (Source: China National Health Commission [36]). NCD: Noncommunicable disease; PMTCT: Prevention of mother-to-child transmission of HIV; MDR-TB: Multidrug-resistant tuberculosis; GP: General Practitioner

The Government in China has explored ways to make health a multi-department priority and ensure cross-sectoral cooperation through a range of mechanisms and institutions. The WHO 2010 Adelaide Statement produced a framework with health promotion as key policy components which has successfully reduced health-related poverty [35]. In 2016, the National Health Commission of China, together with other relevant 14 ministries, issued guidelines for health poverty alleviation programme aimed to break the vicious cycle between poverty and illness by 2020. In 2018, six more concrete actions were proposed to achieve Health-related Poverty Alleviation by 2020. Those actions included (i) improved medical care insurance for the targeted poverty-stricken population; (ii) provision of treatment and health management services covered by serious illness insurance for the poor with serious chronic diseases (which led to the expansion of the spectrum of serious chronic diseases from 9 to 30 diseases); (iii) implementing prevention and control of communicable and endemic diseases using an integrated strategy in poverty-stricken areas aimed at controlling HIV/AIDS, tuberculosis, echinococcosis, schistosomiasis, Kaschin-Beck disease (an endemic type of osteochondropathy) and Keshan disease (cardiomyopathy caused by a combination of selenium deficiency and a mutated *Coxsackie* virus); (iv) improving the delivery capacities in poverty-stricken regions at the county, township and village levels; (v) supporting maternal and child health and health promotion in poverty-stricken region; and (vi) strengthening support systems with priorities in policy making, project allocation, funding and social support to reduce poverty in the areas with most poverty (Fig. 3).

At the technical level, the principal approach was to find gaps related to limited or lack of qualified medical resources and to deliver sufficient and quality reliable health services to local populations. For example, by 2015 there were only 3.66 beds per 1000 population, and 1.28 certified medical doctors or assistants per 1000 population in the 832 poverty-stricken counties in China, the numbers of which are much lower than that of the average numbers at national level [12, 36]. Secondly, precise health-related poverty alleviation approaches were performed at the county, family and individual levels with a focus on the most serious regions, targeted populations, and key diseases, aiming at integrating prevention with treatment by financial assistance through the poverty alleviation programme. Thirdly, detail objectives were identified to ensure provision of primary health care to all poverty-stricken populations, and also to upgrade the capacity of the medical resources and services delivery to the national average level. Fourthly, the three-pronged approaches were implemented by the following steps: (i) reviewing the epidemic trend and financial burden of the diseases among the poor population through a digital information platform; (ii) classifying the poor populations by disease, treatment service and financial protection needed; and (iii) identifying the most important diseases by population and geographical region by mapping for better targeting of medical care services and financial assistance (Fig. 3). In addition, a three-years implementation plan on health-related poverty alleviation between 2018 and 2020 was implemented by the National Health Commission aiming to prioritize eradication of iodine deficiency, skeletal fluorosis and arsenic poisoning caused by coal burning and upgrade disease control with special reference to Kaschin-Beck and Keshan disease, as well as eliminate schistosomiasis as public health problem and effectively control echinococcosis in western China. Good progress on all these fronts has already been noted in the poverty-stricken areas.

## Lessons learnt

The successful lifting 730 million people out of extreme poverty in the past four decades is mainly due to impressive economic growth and coherent policies that favoured improvements in incomes and livelihoods for the poorest of the poor [12, 36]. The major activities of the successful poverty alleviation programme can be summarized as follows:

(i) Through investigation and registration of all poor households and individuals, several key diseases with clear diagnoses and treatment pathways adding financial burdens to the stricken households could be selected. Up to 30 major diseases were covered by the targeted assistance package, including childhood leukaemia, congenital heart disease, tumours, end-stage renal disease to mention a few. With respect to chronic diseases in poor patients, e.g., hypertension, diabetes, tuberculosis and severe mental disorders, family doctors were paid to offer systematic health management.

(ii) By combining disease prevention with treatment along the approaches of the Health Poverty Alleviation programme, both the capacity of health services delivery and financial protection could be improved. Preventive and treatment services for HIV/AIDS, multi drug resistance tuberculosis, Kaschin-Beck disease, Keshan disease and NTDs including schistosomiasis and echinococcosis were among the diseases covered into the targeted disease treatment and subsidy package. The Chinese Government invested in standardized construction of county and township hospitals and village clinics, promoting tiered and integrated health services delivery, supporting hospital-to-hospital assistance between urban and rural areas and encouraging medical college graduates to work in the rural and remote areas in central and western China. In addition, all registered poor people now enjoy a three-tiered financial protection, namely basic health insurance, major disease insurance and medical financial assistance schemes. To support the poor population, out-of-pocket payments were capped at 10% of their health expenditure. For some extremely poor households, out-of-pocket health expenditure was completely covered.

(iii) In order to support the development of better health care in the poverty-stricken regions, the Chinese Government established a strict top-town performance evaluation and accountability mechanism with indicators of poverty alleviation and multi-sectoral cooperation to mobilize various social resources for more precise measures. Many provinces have adopted a mechanism of “one strike and you’re out”. Under this system, a local government's failure to hit poverty alleviation targets cancels out successes against all other performance targets on which it is assessed. Besides fiscal investment, the Government also made important progress in a number of areas identified by researchers as an essential component of poor people’s endogenous development capacities. This includes early childhood development and nutrition, universal health coverage, universal access to quality education and cash transfers to poor families, rural infrastructure, especially roads and electrification and progressive taxation. Private sector and non-governmental organizations, and the communities have also been engaged in the poverty alleviation programme.

Health-related poverty alleviation relies strongly on improving food and nutrition, housing, education, employment and other basic living conditions, which have therefore been incorporated into the systematic, national strategies of the different programmes Health-related Poverty Alleviation, Rural Revitalization and Healthy China 2030. The former programme also promotes other parallel actions tackling key obstacles related to poverty reduction, while the Rural Revitalization Strategy aims to facilitate rural socioeconomic, ecological and cultural development in the post-poverty alleviation period to further consolidate achievements and improve the well-being of the rural population, while the Healthy China 2030 programme acts by improving health infrastructure construction and services delivery in poor counties, thereby providing basic public health services, rehabilitation services and financial protection for the poor.

## Looking forward

The implementation of overarching national strategies in a holistic approach with long-term perspective can theoretically cancel out the impact of negative socioeconomic determinants of health and health-related poverty. However, although the programme on Health-related Poverty Alleviation has already made a great positive impact on socioeconomic development in poor regions, resources such as hospitals beds, doctors and auxiliary staff are still seriously lacking in poor counties which have not been able to deliver sufficient qualitatively reliable services to the population making it difficult to achieve the SDGs health targets in the short term [26]. Therefore, medical services and health insurance and other financial protection systems need to be better aligned. According to the World Bank, the key challenges ahead for China include further improved access of health services for those needing them as well as better data monitoring on poverty and health since those still remaining in poverty, such as the elderly and ethnic minorities, demand even stronger efforts than used so far.

In order to strengthen the health status for all, leaving no one behind and thus achieve the goal of improving the situation for all currently living below the poverty line, the following three actions are recommended: (i) strengthening multi-sectional cooperation and investment coordination during the implementation of health-related poverty alleviation anchored in improving the health services delivery capacities of the rural health facilities, the financial protection capacity to lift out the rural poor with follow-up measures to prevent diseases, maintain health and enhance the productivity abilities; (ii) more intensive and robust research conducive to evidence-based information and its dissemination to decision makers, including research on health systems strengthening in the poor regions, cost-effectiveness analysis and social ethics analysis of priority settings for the decision making in health-related poverty alleviation, and (iii) more actively engagement in global health cooperation and development, such as knowledge sharing and capacity building, to learn from global societies in tackling with the extreme poor with serious illness and incapable to work in the long-run, as well as in generating the experience and lessons from China for other developing countries fighting against health-related poverty.

## Conclusions

The year 2020 marks the end of the major, five-year programme on poverty alleviation initiated by the Chinese Government. Huge progress has already been achieved and the results should now be consolidated to promote further advancement towards the SDGs targets. The challenge in the post-poverty alleviation period is to reach the Healthy China 2030 goal of realizing a world without poverty and endemic diseases. This asks for total elimination of health-related poverty and requires China to provide more assistance to its extremely poor, many of whom struck with serious illnesses, having lost production resources and being in need of long-term health care.

## Data Availability

Not applicable.

## Abbreviations

China: The People’s Republic of China
LMICs: Low and middle income countries
MDGs: Millennium Development Goals
NCDs: Noncommunicable diseases
NTDs: Neglected tropical diseases
SDGs: Sustainable Development Goals
WHO: World Health Organization
